# From ventricle to stoma: a rare case of ventriculoperitoneal shunt extrusion through a colostomy

**DOI:** 10.1093/jscr/rjag518

**Published:** 2026-06-26

**Authors:** Sean Hormozian, Elias Wassel, Neda Salami, Emily Tang, Eric Liou, Jordan Basham, Brandon Woodward

**Affiliations:** Department of Surgery, Arrowhead Regional Medical Center, Colton, CA 92324, United States; Department of Surgery, Arrowhead Regional Medical Center, Colton, CA 92324, United States; Department of Surgery, Arrowhead Regional Medical Center, Colton, CA 92324, United States; School of Medicine, St. George’s University, Grenada, West Indies; Department of Medicine, Noorda College of Osteopathic Medicine, Provo, UT 84606, United States; Department of Surgery, Arrowhead Regional Medical Center, Colton, CA 92324, United States; Department of Surgery, Arrowhead Regional Medical Center, Colton, CA 92324, United States

**Keywords:** ventriculoperitoneal shunt, colonic perforation, adhesiolysis, colostomy

## Abstract

Ventriculoperitoneal shunts are the most common treatment for hydrocephalus but carry rare, potentially life-threatening complications, including bowel perforation. Transmural erosion may occur insidiously without peritonitis due to gradual migration and fibrotic encapsulation, making diagnosis challenging. We report a rare case of transcolonic VP shunt migration with extrusion through a colostomy in a 40-year-old man with spina bifida and a hostile abdominal surgical history. He presented with distal catheter protrusion from his stoma and cerebrospinal fluid drainage into the ostomy appliance. Imaging revealed dense calcification along the shunt tract with proper positioning intracranially. Cerebrospinal fluid cultures grew *Pseudomonas* and vancomycin-resistant *Enterococcus faecium*, requiring culture-directed antimicrobial therapy. Management required multidisciplinary coordination, including emergent shunt externalization, external ventricular drain placement, exploratory laparotomy with adhesiolysis, colotomy repair, and partial hardware removal. This case underscores the extreme rarity and complexity of enteric shunt perforation and the importance of coordinated medical and surgical care.

## Introduction

Ventriculoperitoneal (VP) shunts remain the most widely utilized method of cerebrospinal fluid (CSF) diversion for the management of hydrocephalus. Although generally effective, VP shunts are associated with a significant cumulative lifetime risk of complications. Abdominal complications account for a substantial proportion of failures and include infection, obstruction, catheter migration, and more rarely, perforation [1, 2].

Bowel perforation secondary to shunt placement is an uncommon but potentially life-threatening event with an estimated incidence of only 0.1%–0.7% [3, 4]. The colon is the most frequently involved segment of the gastrointestinal tract [5]. Progressive transmural erosion combined with fibrotic encapsulation may prevent gross peritoneal contamination, allowing the catheter to enter the bowel lumen gradually without causing peritonitis. Consequently, patients may lack abdominal pain or systemic signs. CSF infection may be the first clinical manifestation [3].

The mechanisms underlying shunt-associated colonic perforation are multifactorial. Chronic mechanical irritation, foreign body reaction, and pressure necrosis all contribute. Additionally, long-standing shunts may undergo calcification, resulting in stiffening and fixation within surrounding tissues. Calcified shunts are more difficult to revise and increase the risk of organ injury during explantation [6].

Extrusion of a VP shunt through natural or surgically created orifices represents an extreme manifestation of bowel perforation and is exceedingly rare with very few cases reported in the literature [7]. Reports of shunt externalization through ostomy sites are particularly uncommon and pose unique diagnostic and operative challenges [8]. We present a rare case of transcolonic VP shunt migration with extrusion through a colostomy in a patient with spina bifida and a complex abdominal surgical history.

## Case report

A 40-year-old man with a history of spina bifida, end colostomy for recurrent bowel obstruction, and VP shunt placement at birth (revised at age 13) presented to the ED with protrusion of the distal catheter through his colostomy appliance. Eight months prior to presentation, the patient underwent a diverting colostomy creation for diverticulitis. Six months prior, he required stoma revision with an Altemeier-style repair for prolapse. Operative documentation from an outside institution noted the VP shunt tubing to be freely mobile within the right abdomen at the time of colostomy creation.

On presentation, the patient reported clear fluid draining into the bag consistent with CSF. He denied pain, fever, or neurologic symptoms. Physical examination revealed shunt tubing emerging from the stoma as well as exposed catheter along the anterior abdominal wall beneath an ulcer with expressible purulence. The catheter was immediately clamped where it exited the colostomy to minimize further enteric contamination and ascending infection.

Contrast-enhanced computed tomography of the head, chest, abdomen, and pelvis demonstrated extensive calcification along the shunt tract extending through the chest and abdominal wall. There was no evidence of hydrocephalus on head imaging to suggest that the shunt had occluded. Lumbar puncture revealed CSF cultures growing heavy *Pseudomonas* and vancomycin-resistant *Enterococcus faecium*. Given a documented vancomycin allergy, empiric antimicrobial therapy was initiated with linezolid, metronidazole, and ceftriaxone. Antibiotics were subsequently tailored to meropenem and linezolid based on culture sensitivities in consultation with infectious disease.

**Figure 1:**
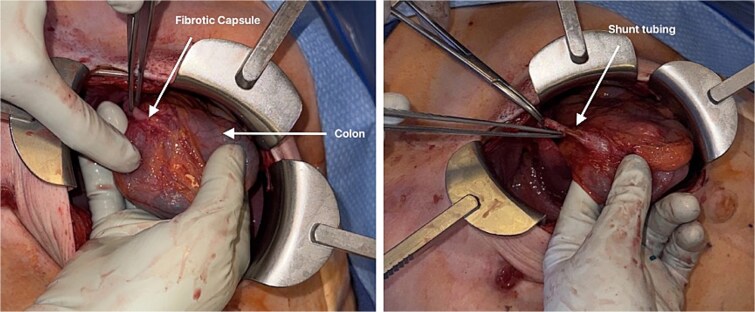
Close-up intraoperative view demonstrating the ventriculoperitoneal (VP) shunt catheter before and after dissection from the surrounding fibrotic capsule. The catheter is visualized penetrating the colonic wall, confirming shunt erosion into the colon. Surrounding inflammatory changes and adhesions are evident at the site of entry.

**Figure 2:**
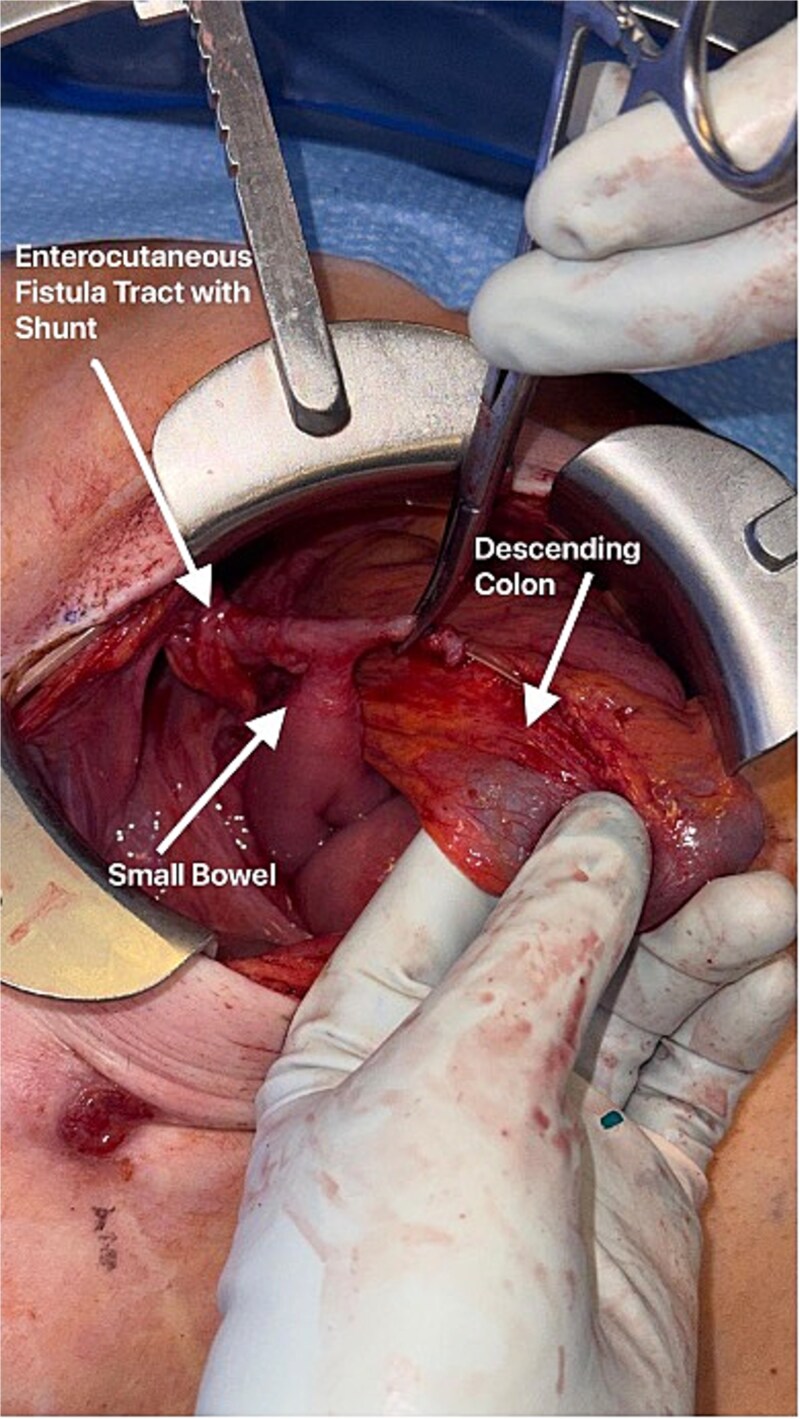
Intraoperative photograph during exploratory laparotomy demonstrating a ventriculoperitoneal (VP) shunt catheter penetrating the left colon. The catheter is densely adherent to the adjacent small bowel along the inferior aspect of the operative field. The shunt-associated enterocutaneous fistula tract is visible on the left side of the image extending toward the abdominal wall.

**Figure 3:**
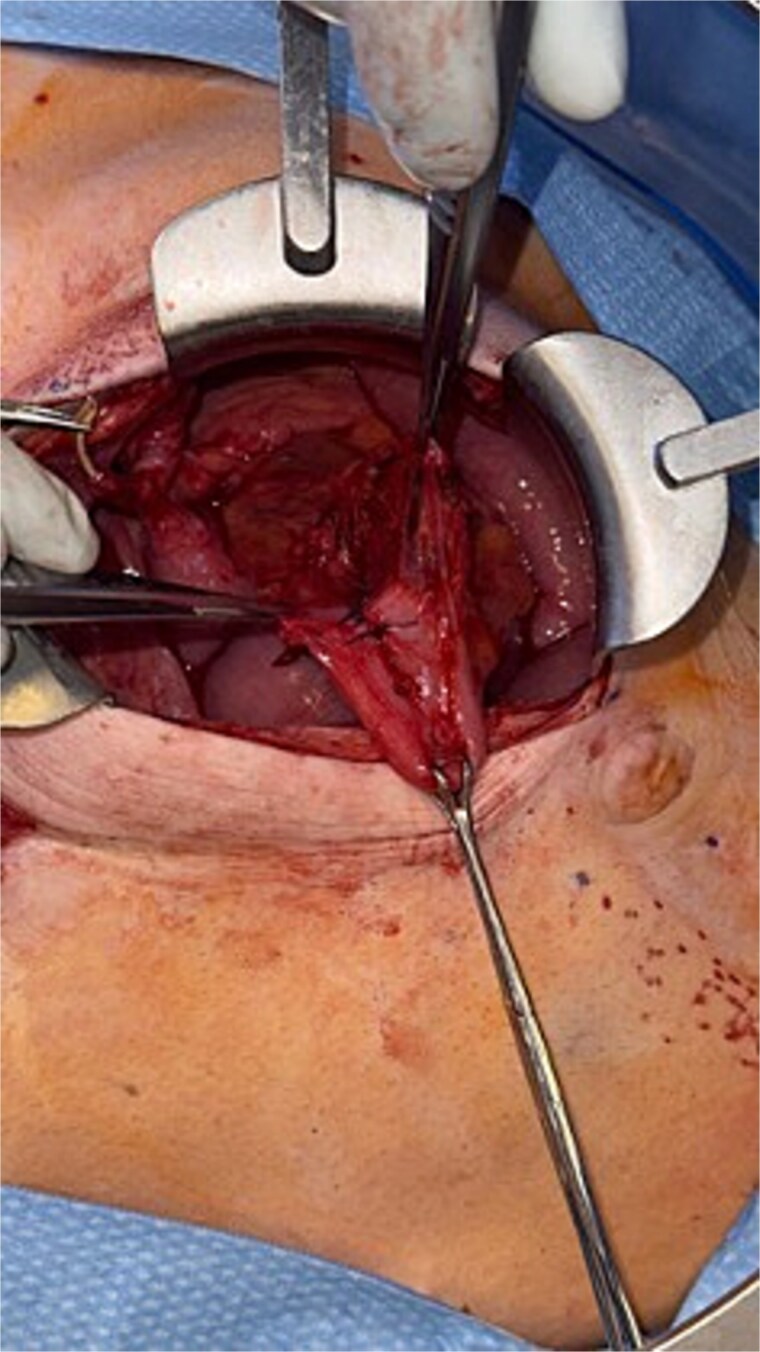
Repair of a small bowel serosal tear incurred from adhesiolysis. The defect is imbricated with interrupted Lembert sutures to reinforce the seromuscular layer.

Neurosurgery promptly externalized the shunt through the chest wall for explantation and placed an external ventricular drain (EVD) to monitor intracranial pressures. The following day, exploratory laparotomy was performed by general surgery. Intraoperatively, the distal catheter was identified entering the colon in the left abdomen within a network of inflammatory changes. The shunt was clamped proximally at the fascia and carefully dissected at the site of perforation (Fig. 1). The catheter was then delivered proximally to the colostomy site; however, there were dense adhesions to the adjacent small bowel that necessitated lysis (Fig. 2). After freeing the shunt from the bowel, it was pulled distally to the enterocutaneous tract and partially explanted. Due to extensive calcification of the proximal tubing within the subcutaneous tissues, complete removal was not feasible; the remaining tract was suture-ligated to prevent further intraabdominal communication. Devitalized colonic tissue was sharply debrided and the defect was closed primarily. A serosal tear was visualized along the small bowel at the site of prior adhesiolysis and repaired (Fig. 3). The abdomen was washed out and closed. Attention was focused to the anterior abdominal wall ulcer where careful dissection was performed to remove further segments of the calcified shunt. The wound was irrigated and packed with iodoform.

The patient’s postoperative course was notable for clearance of CSF cultures and stable neuroimaging. Although shunt revision was initially considered after negative cultures, the patient demonstrated no radiographic or clinical evidence of interval hydrocephalus following EVD removal. Multidisciplinary discussions were held in the event that the patient would need another shunt, and it was recommended by general surgery to wait 4 weeks given the patient’s hostile abdominal conditions. Infectious disease advised shunt replacement was safe after 10 days of negative repeat CSF cultures. The patient was discharged from the hospital in stable condition and remains doing well on follow-up.

## Discussion

VP shunt–associated bowel perforation is rare but carries significant morbidity. In this patient, several factors likely contributed to transcolonic migration. The shunt had been in place for decades and exhibited extensive calcification, which may have promoted fixation to surrounding tissues. Chronic tethering of the catheter to the peristalsing bowel in the setting of a colostomy likely created a static interface that facilitated erosion. This case underscores the importance of early culture acquisition and tailored antimicrobial therapy, particularly given the isolation of resistant organisms.

Infection is one of the most concerning sequelae. Prompt shunt externalization is critical to halt ongoing contamination of CSF. Definitive surgical repair of the bowel injury must account for tissue viability, degree of contamination, and patient-specific anatomic considerations. The presence of calcified hardware may complicate removal given the increased risks of iatrogenic injury with complete explantation.

This case highlights several important considerations regarding adverse risks of VP shunt placement. Bowel perforation may occur decades after shunt placement and can occur without abdominal symptoms. Extrusion through a colostomy is an exceedingly rare, yet possible, presentation. Early recognition of such complications, along with coordinated multidisciplinary care, is necessary in addressing intraabdominal complications of VP shunts.

## References

[1] Bryant MS, Bremer AM, Tepas JJ 3rd et al. Abdominal complications of ventriculoperitoneal shunts. Case reports and review of the literature. Am Surg 1988;54:50–5. 3276260

[2] Ferreira Furtado LM, Da Costa Val Filho JA, Moreira Faleiro R et al. Abdominal complications related to ventriculoperitoneal shunt placement: a comprehensive review of literature. Cureus 2021;13:e13230. 10.7759/cureus.13230 33585146 PMC7877257

[3] Hai A, Rab AZ, Ghani I et al. Perforation into gut by ventriculoperitoneal shunts: a report of two cases and review of the literature. J Indian Assoc Pediatr Surg 2011;16:31–3. 10.4103/0971-9261.74521 21430848 PMC3047774

[4] Snow RB, Lavyne MH, Fraser RA. Colonic perforation by ventriculoperitoneal shunts. Surg Neurol 1986;25:173–7. 10.1016/0090-3019(86)90289-2 3941987

[5] Alves AR, Mendes S, Lopes S et al. Endoscopic management of colonic perforation due to ventriculoperitoneal shunt: case report and literature review. GE Port J Gastroenterol 2017;24:232–6. 10.1159/000454987 29255758 PMC5729945

[6] Salim AD, Elzain MA, Mohamed HA et al. Shunt tube calcification as a late complication of ventriculoperitoneal shunting. Asian J Neurosurg 2015;10:246–9. 10.4103/1793-5482.161321 26396620 PMC4553745

[7] Hasan A, Sharma S, Chopra S et al. Anal extrusion of ventriculoperitoneal shunt: a report of two cases and review of literature. J Pediatr Neurosci 2018;13:8–12. 10.4103/JPN.JPN_97_17 29899765 PMC5982498

[8] Wasadikar PP, Wasadikar AP, Kasbe VP et al. Bowel perforation by ventriculoperitoneal shunt: report of three cases. Int Surg J 2025;12:1521–3. 10.18203/2349-2902.isj20252687

